# Utility of Fibroscan XL to assess the severity of non-alcoholic fatty liver disease in patients undergoing bariatric surgery

**DOI:** 10.1038/s41598-021-93294-6

**Published:** 2021-07-07

**Authors:** Andrew Yang, Melinda Nguyen, Irene Ju, Anthony Brancatisano, Brendan Ryan, David van der Poorten

**Affiliations:** 1 Department of Gastroenterology and Hepatology, Westmead Hospital, Sydney, NSW Australia; 2 Faculty of Medicine, University of Sydney, Sydney, NSW Australia; 3 Sydney Bariatric Clinic, Sydney, NSW Australia; 4 Lakeview Private Hospital, 17-19 Solent Circuit, Northwest, Sydney, NSW 2153 Australia

**Keywords:** Non-alcoholic fatty liver disease, Non-alcoholic steatohepatitis, Obesity, Weight management

## Abstract

Significant weight loss can modify the progression of Nonalcoholic fatty liver disease (NAFLD) with the most convincing evidence coming from bariatric surgery cohorts. Effective ways to non-invasively characterise NAFLD in these patients has been lacking, with high Fibroscan failure rates reported. We prospectively evaluated the utility of Fibroscan using XL-probe over a two-year period. 190 consecutive patients undergoing bariatric surgery were followed as part of their routine care. All patients had Fibroscan performed on the day of surgery and at follow-up a mean of 13 months (± 6.3) later. The majority of patients were female (82%) with mean age of 42. Fibroscan was successful in 167 (88%) at baseline and 100% at follow up. Patients with a failed Fibroscan had higher body mass index (BMI) and alanine transaminase (ALT), but no difference in FIB-4/NAFLD score. Mean baseline Liver stiffness measurement was 5.1 kPa, with 87% of patients classified as no fibrosis and 4% as advanced fibrosis. Mean baseline controlled attenuation parameter was 291, with 78% having significant steatosis, 56% of which was moderate-severe. Significant fibrosis was associated with higher BMI and HbA1c. Significant steatosis was associated with higher BMI, ALT, triglycerides and insulin resistance. Mean follow up time was 12 months with weight loss of 25.7% and BMI reduction of 10.4 kg/m^2^. Seventy patients had repeat fibroscan with reductions in steatosis seen in 90% and fibrosis in 67%. Sixty-four percent had complete resolution of steatosis. Fibroscan can be performed reliably in bariatric cohorts and is useful at baseline and follow-up. Significant steatosis, but not fibrosis was seen in this cohort with substantial improvements post-surgery.

## Introduction

Increasing rates of obesity worldwide are causing a surge in Non-alcoholic fatty liver disease (NAFLD) prevalence with up to 30% of the general population, 80% of the population with obesity^1, 2^ and 90% of bariatric surgery patients with morbid obesity affected^3^. NAFLD is a spectrum of diseases ranging from simple steatosis, to non-alcoholic steatohepatitis (NASH), advanced fibrosis and cirrhosis. Risk factors for the development of NAFLD mirror the metabolic syndrome and include type 2 diabetes mellitus, hypertension, dyslipidaemia, and obesity. Extent of obesity is correlated with fibrosis severity and prognosis in patients with NAFLD compared to non-obese counterparts^4^. Sustained weight loss can modify NAFLD and NASH progression, even leading to resolution, with most convincing evidence in bariatric patients^5^.

Effective non-invasive methods to characterise NAFLD/NASH in bariatric patients have been lacking, with surgeons often relying on liver appearance at operation or liver biopsy to stratify risk. Liver biopsy, although the gold standard for analysing the severity of steatosis and fibrosis^6^, is subject to sampling error and significant risks including bleeding and infection^7, 8^. Fibroscan measures fibrosis by transient elastography and steatosis by controlled attenuation parameter (CAP) and has been validated to stage most liver diseases including NAFLD and NASH. A major limitation for NAFLD however is the failure rate in patients with obesity due to increased subcutaneous fat deposits that reduce the ultrasound signal. Failure rates up to 50% have been reported in morbidly obese and bariatric cohorts^9, 10^.

We investigated the utility of Fibroscan with XL probe for assessing NAFLD and NASH in a bariatric surgery cohort over a two-year period, comparing pre- and post-surgery results to clinical and biochemical measurements. We also report on the outcomes of NAFLD with sustained weight loss in this group.

## Methods

### Patients

One hundred and ninety consecutive patients with obesity undergoing bariatric surgery between June 2017 and May 2019 were prospectively evaluated and followed as part of their routine care. All patients included were over 18 years of age. Those with pre-existing liver disease other than NAFLD/NASH identified on history and routine pre-operative blood tests were excluded from the study, as were those with excess alcohol consumption or with a contraindication to Fibroscan. The study was approved by the Lakeview Private Hospital Medical Advisory Committee with all appropriate guidelines and regulations followed. Informed consent was obtained and all patients deidentified for this study.

### Surgery

Indications for bariatric surgery were the presence of obesity (BMI > 30 kg/m^2^) with either the risk for or current metabolic complications such as insulin resistance, diabetes, hypertension or obstructive sleep apnoea. All surgery was performed by the same surgical team at a single hospital with procedures being either laparoscopic sleeve gastrectomy (LSG) or one anastomosis gastric bypass (OAGB). The choice of procedure was decided by mutual agreement between the surgeon and the patient taking into consideration the risks and benefits of each procedure, the patient's baseline weight and history of comorbid illnesses and type 2 diabetes. All patients were enrolled in a nutrition and lifestyle programme with follow up by the interdisciplinary team (bariatric physician, dietician, bariatric nurse and exercise physiotherapist) at regular intervals for 2 years after surgery. We report blood tests and anthropometric measurements from 6 months post op and repeat Fibroscan with a mean follow-up time of 13 months and median time of 11 months post-surgery (± 6.3 months).

### Laboratory

Patients had a broad panel of blood tests at baseline then at 6 months follow-up. This included full blood count, liver function tests (LFTs), electrolytes, C-reactive protein (CRP), lipid profile, fasting glucose, insulin level, and haemoglobin A1c (HbA1c). NAFLD and Fibrosis-4 (FIB-4) scores were calculated by using parameters of background medical history, body mass index (BMI), aspartate transaminase (AST), alanine transaminase (ALT), platelets and albumin with available formulae previously published^11, 12^. A NAFLD score of > 0.675 and FIB-4 score of ≥ 1.45 was used to determine fibrosis^11, 12^.

### Fibroscan

Liver stiffness measurement (LSM) of fibrosis and Controlled Attenuation Parameter (CAP) measurement of steatosis was performed using FibroScan touch compact 530 (Echosens Paris, France) on the day of surgery. Patients had fasted for at least 6 hours. A single trained operator who had performed over 200 determinations performed all scans using the XL probe, with at least 10 measurements and an interquartile range < 0.2 required for a valid scan. LSM cut-offs of ≤ 6.5 kPa (F0, no significant fibrosis), 6.6 – 8.2 kPa (F1, mild fibrosis), > 8.2 kPa (F2-4, significant fibrosis) and > 9.6 kPa (F3-4, advanced fibrosis) were used. CAP steatosis was graded as: < 248 dB/m (nil significant, grade 0), 249–280 dB/m (mild, grade 1) 281–319 dB/m (moderate, grade 2) and > 320 dB/m for severe steatosis (grade 3) as per recent published guidelines^13^. The diagnosis of NAFLD/NASH was made by the presence of increased CAP and/or LSM scores in the absence of significant alcohol consumption (< 20 g/day for females and < 30 g/day for males) and exclusion of other liver diseases by appropriate history and serological testing.

### Statistical analysis

Data was analysed using IBM SPSS version 21 (Chicago, IL) with statistical significance defined as *p* < 0.05. Continuous variables were reported by mean ± standard deviation and categorical data as frequency and percentages. Groups were compared using Chi-square, student *t*- and ANOVA tests and correlations analysed with Pearson test.

## Results

The baseline characteristics of the 190 patients included in this study are shown in Table 1. The majority were female with a mean age of 42 and from a Caucasian background. Mean weight was 112 kg and BMI 40 kg/m^2^. The majority of patients had insulin resistance or diabetes while 40% had elevated liver function tests (LFTs) and/or dyslipidaemia. The mean NAFLD score and FIB-4 were in keeping with no fibrosis and only 2–3% of all patients had scores suggestive of advanced fibrosis^11^. Eighty percent underwent an LSG with the remainder having an OAGB.

**Table 1:** Baseline characteristics.

Baseline characteristics	Patients (n = 190)
Age, years	42 (± 11)
Females	155 (82%)
Ethnicity	
Caucasian	131 (69%)
Middle Eastern	50 (26%)
Asian	9 (5%)
Weight (kg)	112 (± 20)
BMI (kg/m^2^)	40.2 (± 6.6)
30–34.9	34 (18%)
≥ 40	91 (48%)
≥ 50	16 (8%)
Surgery type	
Sleeve gastrectomy	152 (80%)
One anastomosis gastric bypass	38 (20%)
ALT > 30 IU/L	76 (40%)
GGT > 30 IU/L	70 (37%)
Cholesterol ≥ 5.5 mmol/L	64 (34%)
Triglycerides ≥ 2.0 mmol/L	43 (23%)
IR/IFG	140 (74%)
Diabetes	23 (12%)
Hypertension	39 (21%)
NAFLD score > 0.675 (F3-4)	5 (2.6%)
FIB-4 > 1.45 (F3-4)	6 (3.2%)

*BMI* Body Mass Index, *ALT* Alanine aminotransferase, *GGT* Gamma glutamyltransferase, *IR* Insulin resistance, *IFG* impaired fasting glucose.

Results are reported as frequency (percentage) or mean (SD) as appropriate.

### Factors associated with liver fibrosis and steatosis by Fibroscan

The majority of patients had significant liver steatosis (≥ Grade 1; 78%) with a mean CAP score of 291.4 dB/m and over a third with severe steatosis (Grade 3; Table 2). Only 6% had significant fibrosis (≥ F2) however, with a mean LSM score of 5.1 kPa. Out of these, 7 patients had advanced fibrosis (F3-4) (Table 2), with only 1 patient having intraoperative cirrhosis. As expected, weight and BMI were strongly associated with both steatosis and fibrosis (*p* < 0.05). Significant steatosis was associated with higher ALT (*p* < 0.05), triglycerides (*p* < 0.05), insulin level (*p* < 0.001), fasting glucose (*p* < 0.05) and HbA1c (*p* < 0.01). Significant fibrosis was associated with a higher HbA1c only (*p* < 0.05). NAFLD score correlated with severity of fibrosis (*p* < 0.01), but FIB-4 score did not (*p* = 0.1). Neither scores had statistical associations with the CAP reading.

**Table 2:** Baseline Fibroscan results.

Fibroscan Data	Baseline (n = 167)
Steatosis CAP (dB/m)^a^ (dB/m)	291.4 (± 58.8)
Grade 0	37 (22%)
Grade 1	36 (22%)
Grade 2	35 (21%)
Grade 3 (severe)	59 (35%)
Significant steatosis (≥ S1)	130 (78%)
Fibrosis LSM (kPa)^b^	5.1 (± 1.9)
Stage 0 (≤ 6.5 kPa)	145 (87%)
Stage 1	12 (7%)
Stage 2	3 (2%)
Advanced Fibrosis (3–4)	7 (4%)
Significant fibrosis (≥ F2)	10 (6%)

Results expressed as mean (± SD) or frequency (percentage).

^a^CAP = Controlled attenuation parameter steatosis grades: 0 (nil significant) < 248 dB/m; 1 (mild) 248–280 dB/m; 2 (moderate) = 281-319 dB/m; 3 (severe) > 320 dB/m.

^b^LSM = Liver stiffness measurement fibrosis stages: 0 (no significant fibrosis) ≤ 6.5 kPa; 1 (mild) 6.6–8.2 kPa; 2 (significant) > 8.2–9.6 kPa; 3–4 (advanced) > 9.6 kPa.

### Fibroscan success

Fibroscan was successful in 167 out of 190 patients (87.9%) at baseline, and 100% at follow-up (≥ 6 months post-surgery), with 70 patients receiving a follow-up Fibroscan within the study period (Supplementary Fig. 1). Unsuccessful Fibroscan measurements were associated with increased weight (129 vs. 109 kg, *p* < 0.01) and BMI (46.2 vs. 39.3 kg/m^2^, *p* < 0.01), but not with LFTs, platelets, NAFLD score or FIB-4, meaning patients with significant fibrosis were not missed.

### Changes after bariatric surgery

Mean time to follow-up fibroscan was 13 months, median time of 11 months (± 6.3 months) with weight loss of 25.2% (p < 0.01) and BMI reduction of 10.4 kg/m^2^ (*p* < 0.01). There were significant reductions post-surgery in GGT (36 vs. 22.8; *p* = 0.001), fasting glucose (5.7 vs. 4.9; *p* < 0.001), insulin level (20.9 vs. 8.5; *p* < 0.001), HbA1c (5.7% vs. 5.2%; *p* < 0.001), total cholesterol (5.3 vs. 4.8; *p* < 0.001), triglycerides (1.6 vs. 1.2; *p* < 0.001) and in the NAFLD score (−1.636 vs. −2.123, *p* < 0.001). There was no association between type of surgery (LSG or OAGB) with changes in weight, BMI, steatosis or fibrosis.

**Figure 1:**
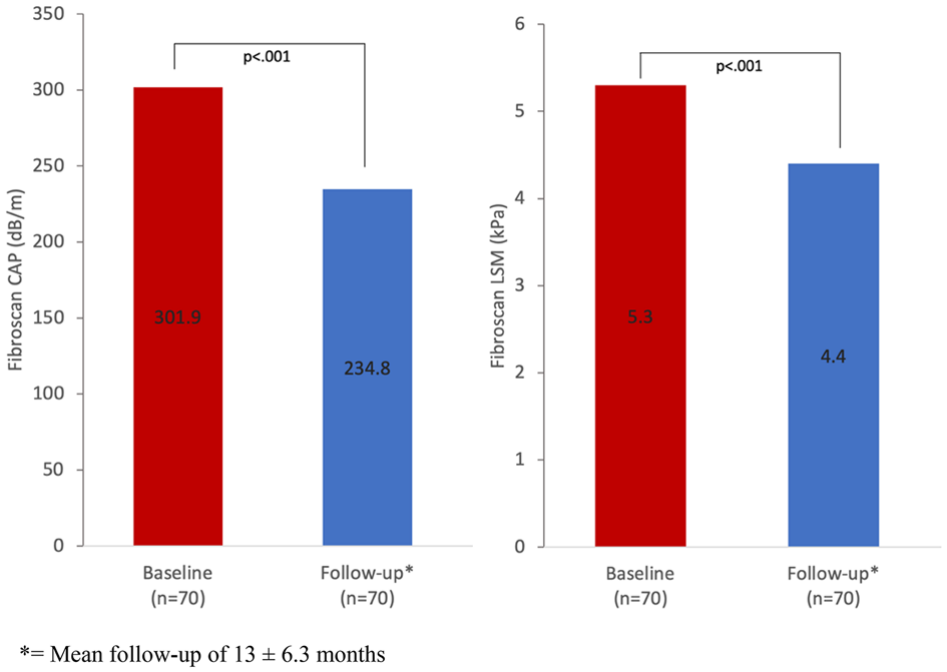
Changes in hepatic steatosis (CAP) and fibrosis (LSM) by fibroscan post surgery.

**Figure 2:**
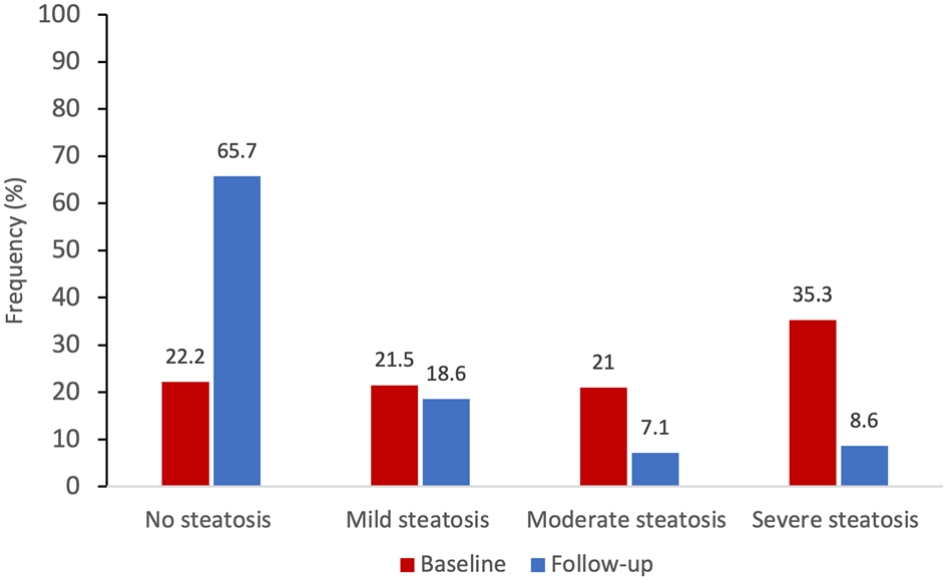
Comparison of CAP steatosis scores at baseline and follow-up post-surgery.

Seventy patients had both a baseline and follow-up Fibroscan. There was significant improvement in steatosis and fibrosis measures post-surgery, with a mean reduction of CAP from 301.9 to 234.8 dB/m (*p* < 0.001) and of LSM from 5.3 to 4.4 kPa (*p* < 0.001, Table 3 and Fig. 1). Overall 90% of patients had a reduction in CAP and 64% had complete resolution of steatosis. By steatosis grades, 75.7% of patients had an improvement of one or more grades, 18.6% had no change and 5.7% had worsening (Fig. 2). Advanced steatosis was eradicated in all but 5 patients (Table 3). The 4 patients with an increase in steatosis after surgery were older (55 vs. 44 years) and had a lower average weight loss (21.9% vs. 27.1%). None of these patients had significant fibrosis before or after surgery, meaning the minor increase in steatosis was unlikely to be clinically significant.

**Table 3:** Fibroscan results pre and post-surgery.

Fibroscan data	Baseline (n = 70)	Follow-up (n = 70)	*p* value
Mean CAP Steatosis (dB/m)	301.9 (± 52.9)	234.9 (± 53.0)	< 0.001
Significant steatosis (≥ 248 dB/m)	61 (87%)	24 (34%)	< 0.001
Severe steatosis (≥ 320 dB/m)	24 (34%)	5 (7%)	< 0.001
Mean LSM Fibrosis (kPa)	5.3 (± 1.9)	4.4 (± 1.2)	< 0.001
Significant fibrosis (> 8.2 kPa)	5 (7%)	0	< 0.05
Advanced fibrosis (> 9.6 kPa)	4 (6%)	0	NS

Sixty seven percent of patients had reduced LSM post-surgery with males having a greater reduction than females (−2.0 vs —0.6 kPa, *p* < 0.01). By fibrosis stage, 10% had improvement, 88.6% had no change and one patient had worsening. No patients had significant or advanced fibrosis (> 8.2 kPa) at follow-up (Table 3). The single patient with increased fibrosis stage was 26 years old, had LSG surgery and lost 23.8% bodyweight at the time of repeat scan. LSM increased from 5.8 to 8.1 kPa meaning a minimal change from fibrosis stage 0 to stage 1.

There was no difference in comorbidities and pre-op or post-op BMI between those who did not have a follow up Fibroscan (n = 97) and those that did. Baseline steatosis measured by CAP, NAFLD and FIB-4 were all lower in this group.

## Discussion

This study of 190 patients undergoing bariatric surgery highlights the high rates of underlying NAFLD and the utility of Fibroscan in stratifying patient risk for significant liver disease. The majority of patients in our cohort had significant hepatic steatosis by fibroscan, with a third having severe steatosis and 4% advanced fibrosis or cirrhosis. As expected, increasing weight and BMI correlated with the presence of steatosis or fibrosis and hence a diagnosis of NAFLD. Steatosis severity was associated with rising ALT and metabolic markers including triglycerides, insulin, fasting glucose and HbA1c. Fibrosis severity was only correlated with HbA1c. These findings are comparable with previous studies that performed liver biopsies at time of bariatric surgery^3, 14^.

Fibroscan has previously been considered difficult in a morbidly obese population with unreliable results or scan failure in up to 50% of patients^10, 15^. This has led to higher rates of liver biopsy than may be necessary. Using the Fibroscan XL probe and an experienced operator we had a high success rate of 88% at baseline pre-op and 100% at follow-up, even with mean BMI in these groups being 40.2 and 28.7, respectively. A number of recent reports using the XL probe have shown similar high success rates^15–18^ confirming the utility of transient elastography even in extreme obesity. Fibroscan failure is almost always caused by excess subcutaneous fat overlying the right chest wall which increases the skin to liver capsule distance^10, 19^. In line with this, increasing weight and BMI predicted scan failure in our group. There was no association however, between unsuccessful fibroscan measurements and liver enzymes (AST, ALT, GGT), platelet count, NAFLD score or FIB-4 score, suggesting that patients with advanced fibrosis were not missed with an unsuccessful fibroscan.

The positive effects of bariatric surgery induced weight loss on cardiometabolic risk are well established^20, 21^ and in line with this we demonstrated improvements in insulin resistance, HbA1c and lipids. The role of bariatric surgery in modulating the disease trajectory of NAFLD is less well established with limited numbers of reports showing improved disease markers or histology^22, 23^. A recent trial involving paired liver biopsies and fibroscan pre and one-year post surgery provided positive results but with some cautions^18^. In the 58 patients studied, all histological parameters were improved on average, with reversal of cirrhosis in two of three effected patients and resolution of NASH in one third. There was worsening of fibrosis, however, in 17%, 10% of which occurred in patients with no baseline fibrosis. De novo steatosis was seen in 5% of patients. The only clear association with these deteriorations were increased patient age. These results are supported by a meta-analysis of 32 cohort studies^23^ where the resolution of NAFLD in many had to be tempered by worsening histology in a minority without clear cause. Fibroscan LSM and CAP scores in the study by Agarwal and colleagues correlated well with liver histology both and pre and post surgery, although cutoffs for steatosis grades and fibrosis stages were higher pre-operatively.

Two other studies have looked at the utility and accuracy of fibroscan outcomes post bariatric surgery^14, 16^ and demonstrated NAFLD improvement on follow-up^16^. Compared to our study however, patient numbers were smaller and the NAFLD fibrosis score or FIB-4 were not utilised. Follow up fibroscan around one-year post surgery in our cohort showed significant improvements in steatosis and fibrosis for almost every patient. Close to two thirds of patients rescanned had complete resolution of steatosis and all had resolution of significant fibrosis (≥ F2), demonstrating the potential reversibility of NAFLD with significant weight loss. Anecdotally, patients found the repeat fibroscan a very positive and motivating experience, demonstrating directly an improvement in their liver health to go along with the weight loss achieved.

A reassuring finding of this study was the low level of significant fibrosis or worsening of NAFLD found. Six percent of patients had fibroscan readings suggestive of fibrosis stage 2 or above and this correlated with the non-invasive NAFLD and FIB-4 scores. Previous studies have reported up to 27% having significant fibrosis (grade 2–3) and 10% with advanced fibrosis or cirrhosis^24, 25^. This discrepancy may be explained by the relatively young age of our cohort (mean 42 years) and low incidence of diabetes mellitus (12%), even though mean BMI was 40.2 kg/m^2^. Only one patient in our cohort had an increase in fibrosis from stage 0 to stage 1 despite 23% weight loss and young age. Four patients had an increase in steatosis, but in the context of no fibrosis before or after surgery the overall impact of this was considered minimal. Taken together, this suggests that bariatric cohorts with similar characteristics can be managed without liver biopsy. Fibroscan can then be used in older patients, those with less well controlled diabetes, elevated LFTs and the highest BMI.

A limitation of this study was the relatively small number of patients who agreed to follow up fibroscans, in total 70 out of 190. This in part reflects the asymptomatic nature of NAFLD, with patients not perceiving any problem and already aware of biochemical and metabolic improvements on blood tests. Despite this, we were able to show substantial and important improvements in NAFLD in the majority. Our follow-up time of 12 months provides short to medium term data in line with other recent studies^18^. Longer-term data showing the impact of bariatric surgery on NAFLD/NASH beyond 12 months will be important to guide the use of this treatment modality.

In conclusion, fibroscan with XL probe is a useful tool to assess NAFLD and NASH in bariatric surgery patients with obesity and can be performed reliably and safely both pre- and post-operatively. We showed substantial improvements of hepatic steatosis and fibrosis at follow up in conjunction with significant weight loss and demonstrated the reversibility of NAFLD following bariatric surgery.

## Supplementary Information

Supplementary Information.
