# Supplementary figures and images for: Utility of Fibroscan XL to assess the severity of non-alcoholic fatty liver disease in patients undergoing bariatric surgery
Source: Sci Rep. 2021 Jul 7;11:14006. doi: 10.1038/s41598-021-93294-6 (PMC8263818; doi:10.1038/s41598-021-93294-6)

**Supplementary Figures**


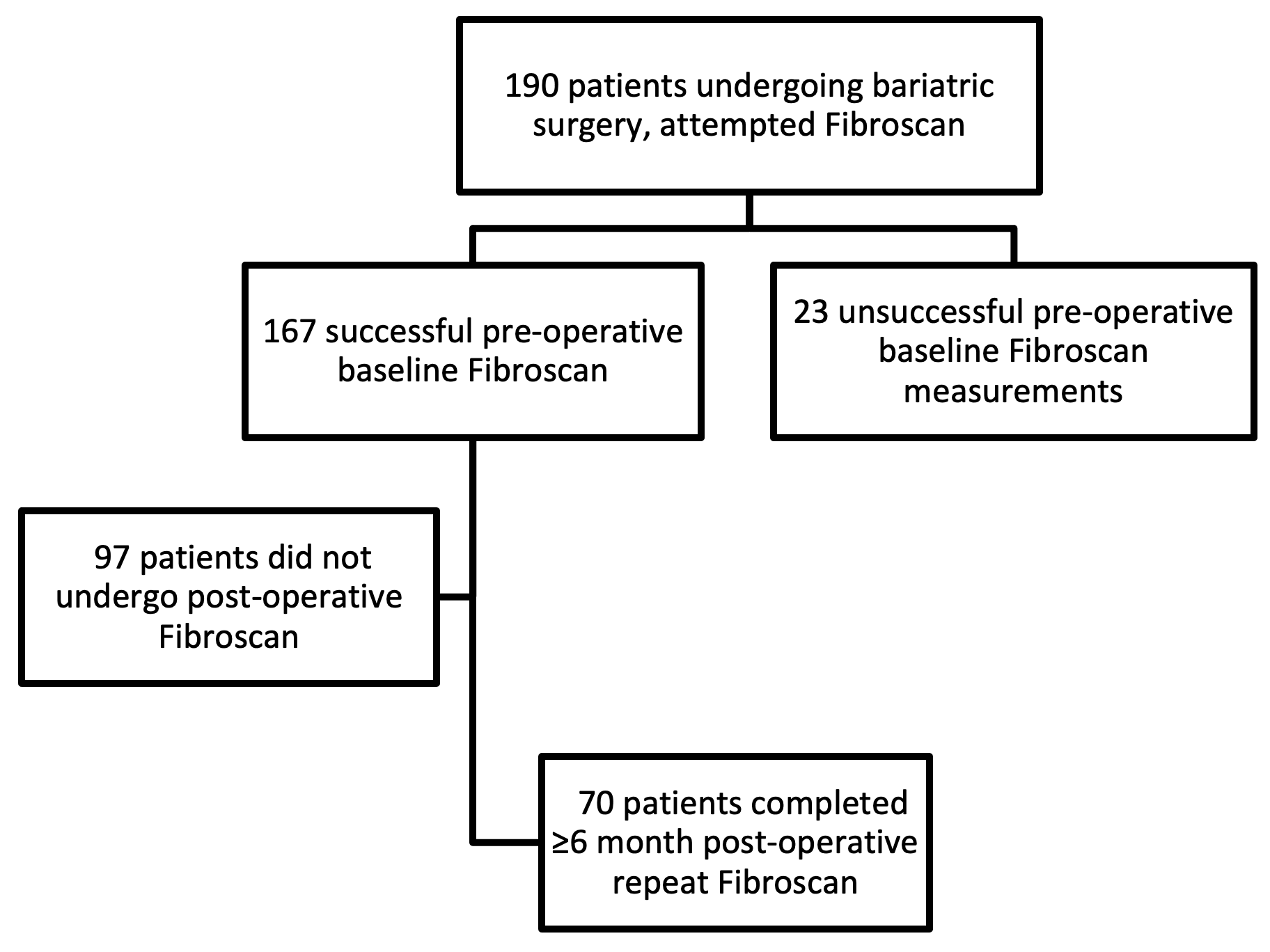


Supplementary Figure 1. Flow-chart of patients

Supplement: Supplementary file 1 — Supplementary Information. [file 41598_2021_93294_MOESM1_ESM.docx]
